# Hidden effects of habitat restoration on the persistence of pollination networks

**DOI:** 10.1111/ele.14081

**Published:** 2022-08-25

**Authors:** Marilia P. Gaiarsa, Jordi Bascompte

**Affiliations:** ^1^ Department of Evolutionary Biology and Environmental Studies University of Zurich Zurich Switzerland; ^2^ School of Natural Sciences University of California, Merced Merced California USA

**Keywords:** dynamical model, invasive species, modularity, nestedness, network centrality, species interactions

## Abstract

Past and recent studies have focused on the effects of global change drivers such as species invasions on species extinction. However, as we enter the United Nations Decade of Ecosystem Restoration the aim must switch to understanding how invasive‐species management affects the persistence of the remaining species in a community. Focusing on plant‐pollinator interactions, we test how species persistence is affected by restoration via the removal of invasive plant species. Restoration had a clear positive effect on plant persistence, whereas there was no difference between across treatments for pollinator persistence in the early season, but a clear effect in late season, with higher persistence in unrestored sites. Network structure affected only pollinator persistence, while centrality had a strong positive effect on both plants and pollinators. Our results suggest a hidden effect of invasive plants—although they may compete with native plant species, invasive plants may provide important resources for pollinators, at least in the short term.

## Graphical abstract

As we enter the United Nations Decade of Ecosystem Restoration the aim must switch to understanding how invasive‐species management affects the persistence of the remaining species in a community. We found that restoration via the removal of invasive plant species has a clear positive effect on plant persistence, whereas for pollinators persistence was higher in unrestored sites in the late season. Our results highlight that network structure may be a useful tool in restoration studies to assess the impact of restoration practices on population dynamics and emphasize a hidden effect of invasive plants‐‐‐although they may compete with native plant species, invasive plants may provide important resources for pollinators.

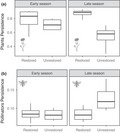

## INTRODUCTION

We are currently undergoing an unprecedented biodiversity crisis (Barnosky et al., 2011; Johnson et al., 2017). The latest IPBES report (IPBES, 2019) starkly illustrates how catastrophic human impacts on biodiversity have become. In addition to climate change and land‐use change, invasive species are one of the most detrimental global change drivers, having increased seven‐fold over the last 75 years (Fricke & Svenning, 2020). Given the ubiquity of invasive species across the globe (Fricke & Svenning, 2020; IPBES, 2019; Traveset & Richardson, 2020) and the increased fragility of communities to their presence (Suding et al., 2016; Tylianakis & Morris, 2017), understanding the effects that invasive species have on ecological communities is of paramount importance.

Ecological restoration is such an effective solution to help reverse the biodiversity crisis (Cariveau et al., 2020; Miller et al., 2017) that the United Nations have declared 2021−2030 the “Decade of Ecosystem Restoration”. The main goal of ecological restoration is to re‐establish the structure and composition of a community that has been damaged so that ecological functions and processes, as well as community dynamics, can be somewhat recovered (Clewell et al., 2004; Clewell & Aronson, 2012; Forup et al., 2008). Ecological restoration can be implemented through different management actions that can be used to address multiple environmental threats. Across biological contexts, ecological restoration has been shown to be essential to mitigate climate change (IPCC, 2019) and habitat fragmentation (reviewed in Cariveau et al., 2020), buffer species extinction (IPBES, 2019), and improve the provision of ecosystem services (Chazdon, 2008; Forup et al., 2008; Kaiser‐Bunbury et al., 2017; Shackelford et al., 2013). Although plant communities have been the focus of the majority of research (Brudvig, 2011; Kaiser‐Bunbury & Simmons, 2020; Ruiz‐Jaen & Mitchell Aide, 2005; Vilà et al., 2011), a growing number of studies have explored the effects of restoration on animals (Cariveau et al., 2020; Guyton et al., 2020; Noreika et al., 2019; Vilà et al., 2011). While the recovery of native species can sometimes suppress invasive species (e.g. Guyton et al., 2020), human intervention is more often required. One way to restore ecosystems is to remove exotic and/or invasive species (Clewell et al., 2004; Clewell & Aronson, 2012) under the assumption that native species will benefit from a competition release. For example, the presence of an exotic nectar‐rich plant species in New Zealand appears to have led to a decrease in the pollination, seed production, and density of a native shrub (Anderson et al., 2011). However, because it is both financially and logistically difficult to collect long‐term data on population persistence, the knowledge of the effects of restoration on population persistence is limited. To fully understand the effectiveness of restoration via invasive‐species removal, it is necessary to assess not only how the eradication of invasive species affects ecosystem function (Cariveau et al., 2020; Forup et al., 2008; Miller et al., 2017) and the diversity and abundance of the remaining species (Kaiser‐Bunbury et al., 2017), but also population persistence in the long‐term (Miller et al., 2017; Shackelford et al., 2013).

While most restoration studies have focused on species per se, species interactions are the ones responsible for maintaining ecosystem function and providing ecosystem services, and often disappear faster than species themselves (Newbold et al., 2015; Tylianakis et al., 2010). Network theory provides powerful tools for understanding ecological systems (Bascompte & Jordano, 2007) and enables us to consider the community context of ecosystem restoration, evaluating ecological assemblages in terms of both species and their interactions (Frost et al., 2019; Kaiser‐Bunbury & Simmons, 2020; Memmott, 2009; Traveset & Richardson, 2020). Although our understanding of the consequences of invasive species in mutualistic networks has increased in recent years (Emer & Timóteo, 2020; Kaiser‐Bunbury et al., 2017; Traveset & Richardson, 2014; Tylianakis & Morris, 2017), it remains unclear how invasive species affect the persistence of native species within the community. Overall, evidence suggests that the presence of invasive plant species negatively affects native plant species (Levine et al., 2003; Vilà et al., 2011). We can thus expect that invasive‐species removal is beneficial to native plant species (e.g. Anderson et al., 2011). However, invasive plants may also increase ecosystem productivity so their removal might have positive bottom‐up impacts (Russell & Kaiser‐Bunbury, 2019; Vilà et al., 2011). Moreover, the disturbance generated by the removal of invasive species may have negative effects on native species (Heleno et al., 2010; Traveset & Richardson, 2020), which may hinder ecosystem's ability to withstand further perturbations even after an ecosystem has been restored. Thus, understanding the critical relationship between restoration practices via invasive‐species removal and the persistence of the remaining species in the community (Rodewald et al., 2015; Shackelford et al., 2013) will broaden the understanding of species responses to different global change drivers while also helping to devise better management tools for protection and recovery of both species and ecosystems.

By evaluating descriptive measures of network structure, one can also gain insights into the mechanisms behind species invasion and restoration success (Emer & Timóteo, 2020; Rodewald et al., 2015). For example, nested networks—a pattern in which a core group of generalists interacts with both specialists and generalists (Bascompte et al., 2003)—may be more vulnerable to invasion (Frost et al., 2019; Traveset & Richardson, 2020). A nested structure results in a greater redundancy in interaction patterns, which, in contrast, has been attributed to an increase in community robustness to species loss (Lever et al., 2014; Rohr et al., 2014; Thébault & Fontaine, 2010). Another commonly used metric to characterise interaction networks is modularity, a pattern in which a group of species interacts more among themselves than with species outside of the group (Olesen et al., 2007). The presence of modules may increase species persistence by limiting the spread of perturbations (Stouffer & Bascompte, 2011) while also hindering the establishment of invasive species (Emer & Timóteo, 2020). However, modularity may decrease following invasion (Albrecht et al., 2014). Moreover, network theory can be used to assess not only changes in the overall community structure, but also changes at the species level. For example, following invasion, non‐native species usually displace or outcompete native species (Emer & Timóteo, 2020) and may assume highly generalist positions in their networks (Arroyo‐Correa et al., 2020; Olesen et al., 2002; Traveset & Richardson, 2020), which could increase heterospecific pollen deposition for native species (Johnson & Ashman, 2019). Thus, exploring network metrics at the species level enables us to characterise resource overlap and diet breadth, which have also been linked to community stability and species persistence (Benadi et al., 2012; Rodewald et al., 2015).

Here, we address how restoration via invasive‐species removal affects community resilience by combining dynamic models of species persistence in ecological networks with experimental data on the mutualistic interactions between plants and pollinators at sites where invasive plant species have either been removed (“restored”) or maintained (“unrestored”; Kaiser‐Bunbury et al., 2017). We hypothesise that (1) both plant and pollinator persistence would be higher in restored sites than in unrestored sites. Moreover, given that previous work showed that species persistence is affected by network structure (Bastolla et al., 2009; Rohr et al., 2014; Thébault & Fontaine, 2010) and the expectation that persistence would be higher in restored sites, we also hypothesised that (2) the effect of network structure on persistence would differ between restored and unrestored sites. Finally, we also explored if (3) more central species would have greater persistence because they have access to a wider range of resources (wider diet breadth) and are thus better buffered than more peripheral species to the loss of interaction partners, and whether this relationship would be different between restored and unrestored sites. By combining dynamic population models and ecological restoration via invasive‐species removal, our results broaden the understanding of how species invasions alter ecological communities and ecosystem services—essential if we are to mitigate the negative effects of different extinction drivers and ensure the provision of ecosystem services.

## MATERIALS AND METHODS

### Empirical data

We used 64 plant‐pollinator networks from the island of Mahé, Seychelles previously published by Kaiser‐Bunbury et al. (2017). Briefly, the dataset is composed of eight sites (inselbergs) distributed across two treatments: (1) restored sites—where invasive plants were removed prior to data collection (between November 2011 and February 2012)—and (2) unrestored sites, left unaltered (Kaiser‐Bunbury et al., 2017). Thus, here we refer to restoration as the practice of removing invasive species from a site. Sites were spatially independent of each other, and interactions were recorded monthly throughout the complete flowering season (between September 2012 and April 2013), resulting in 64 networks (Kaiser‐Bunbury et al., 2017). Each network depicts the interaction frequency (number of interactions) between plants and their pollinators. The dataset contains 144 different pollinators that were identified to the highest taxonomic unit, encompassing species of bees and wasps (Hymenoptera), moths and butterflies (Lepidoptera), flies (Diptera), beetles (Coleoptera), as well as two bird species (Nectariniidae, Pycnonotidae) and three lizard species (Gekkonidae, Scincidae) (Kaiser‐Bunbury et al., 2017). A detailed description of species and data collection methods can be found in Kaiser‐Bunbury et al. (2017). The Seychelles experience a humid, tropical climate, marked by a dry and wet season (Lawrence, 2004), resulting from the effect of monsoons. Thus, in addition to analysing the month‐level data, we explored the effect of seasonality on species' persistence in this system. We compared the beginning and the end of the monsoon season by aggregating monthly networks into early season (September to December), and late season (January to April), yielding 16 networks (two networks per site, eight networks per treatment). For all network analyses, when the network was compartmentalised we only included species present in the largest component. Below, we present all the results for both the monthly data (*N* = 64 networks, eight per site) and the seasonal data (*N* = 16 networks, two per site).

### Dynamic model

To study the population dynamics of species in each network, we used the dynamic model proposed by Bastolla et al. (2009), composed of a set of differential equations that describe the coupled dynamics of plants (P) and pollinators (A). We modelled changes in abundance over time for species i as:

(1) $$\begin{array}{c} \frac{{{\mathrm{dS}}_{i}}^{\left(P\right)}}{\mathrm{dt}}={\alpha_{i}}^{\left(P\right)}{S_{i}}^{\left(P\right)}-\sum_{\mathrm{j\varepsilon P}}\beta_{i}j^{\left(P\right)}{S_{i}}^{\left(P\right)}{S_{j}}^{\left(P\right)}\\ +\sum_{\mathrm{k\varepsilon A}}\frac{\gamma_{\mathrm{ik}}^{\left(P\right)}{S_{i}}^{\left(P\right)}{S_{k}}^{\left(A\right)}}{1+h^{\left(P\right)}\sum_{\mathrm{l\varepsilon A}}\gamma_{\mathrm{il}}^{\left(P\right)}{S_{l}}^{\left(A\right)}}. \end{array}$$

Equation 1 describes the dynamics of plant species i as a function of their intrinsic growth rate ($\alpha_{i}$), interspecific competition with plant j ($\beta_{\mathrm{ij}}$), intraspecific competition ($\beta_{\mathrm{ii}}$) and mutualistic effects based on the interaction strengths with pollinator k ($\gamma_{\mathrm{ik}}$). The mutualistic benefit $\gamma_{\mathrm{ik}}$ depends on the per capita effect of animal k on plant i. We assumed that intraspecific competition $\beta_{\mathrm{ii}}$ is stronger than interspecific competition $\beta_{\mathrm{ij}}$ to prevent species from outcompeting each other (Bastolla et al., 2009; Lever et al., 2020). As in Bastolla et al. (2009), we also assumed a type II functional response (denominator term) in which species are limited by their capacity to process resources ($h^{\left(P\right)}$); as a result, interaction frequency saturates when resource availability is high. The equation describing the dynamics of pollinators is the same as equation (1), but with the indices (P) and (A) exchanged.

We ran the dynamic model for each of the 64 monthly networks (month‐level data), as well as for the early‐ and late‐season data (16 networks; season‐level data). For all analyses presented in the main text, we limited the pollinator guild to invertebrate species (139 out of the 144 species) because of the order‐of‐magnitude differences in invertebrate and vertebrate longevity, and the model assumption that animals (pollinators) are completely dependent on plants for their survival, which may not be true for the five vertebrate species present in the original dataset (Supporting Information). However, results are qualitatively similar when vertebrates are included in the models (Supporting Information, Figs S2–S5).

Following previous studies (Bastolla et al., 2009; Lever et al., 2020; Saavedra et al., 2011), we used the following parameter values: intrinsic growth rates $\alpha_{i}$ were drawn uniformly from the interval $\left[0.85,1.1\right]$; values for intraspecific competition $\beta_{\mathrm{ii}}$ were drawn uniformly from the intervals $\left[0.99,1.01\right]$ and values for interspecific competition $\beta_{\mathrm{ij}}$ from $\left[0.22,0.24\right]$; the per capita effects of mutualistic interaction, γ were drawn uniformly from the interval $\left[0.19,0.21\right]$; handling time h was fixed and set to 0.1; initial abundance densities S were drawn uniformly from the interval $\left(0,1\right]$ (Bastolla et al., 2009; Lever et al., 2020; Saavedra et al., 2011). Other parameter values did not alter our results (Supporting Information, Figs S6–S8). We performed simulations in R (version 4.1) (R Core Team, 2021) by integrating the system of ordinary differential equations using the ode function from the deSolve package (version 1.28) (Soetaert et al., 2010), with small integration steps. We ran $10^{3}$ simulations for each network in the month‐level and season‐level datasets. We considered a species to be extinct when their abundance density $S_{i}$ at the end of a simulation run was $<10^{-10}$ (Bastolla et al., 2009). For the species‐level analyses (see below), for each species at each network we calculated the proportional persistence across all simulations runs (i.e., when abundance density $>10^{-10}$). Similarly, for the network‐level analyses, we calculated proportional persistence as the mean proportion of all species that persisted across all simulations runs.

### Network metrics

For each network at the month‐level (*N* = 64) and season‐level (*N* = 16) datasets, we characterised community structure using two common network‐level metrics: nestedness and modularity. We used the bipartite package (Dormann et al., 2008, 2009) to calculate nestedness (NODF), and the igraph package (Csardi & Nepusz, 2006) to calculate modularity using the spin‐glass model and simulated annealing algorithm.

Likewise, for each species in month‐level (*N* = 64) and season‐level (*N* = 16) network, we also calculated two centrality metrics as a measure of resource overlap and diet breadth: weighted closeness and specialisation (d’). Weighted closeness centrality measures the number of shortest paths between a focal species and every other species in the network—the closer a species is to all other species in the network, the more central a species is and thus, the larger the closeness centrality, resulting in greater resource overlap (González et al., 2010). Specialisation, in turn, can be seen as a proxy for niche breadth, and is based on a null model approach and considers how much observed interaction frequencies deviate from what is expected based on partner availability (Blüthgen et al., 2006). The greater the value of specialisation, the more specialised in a resource a species is. We calculated weighted closeness centrality and specialisation (d’) using the bipartite package (Dormann, 2011; Dormann et al., 2008).

### Statistical analyses

We used generalised linear mixed‐effects models to test the effect of restoration status of each site on species persistence at the month level. Our model included month, restoration status, and their interaction as fixed effects and included by‐site random intercepts. We assumed a beta‐error distribution because our response variable, mean proportion species persistence, is bounded between 0 and 1. Similarly, to test for effects of restoration status and seasonality on species persistence, we considered season, restoration status and their interaction as fixed effects and included by‐site random intercepts. To further unravel the effects of seasonality, we also used a generalised linear model with a beta‐error distribution to test for the effect of early and late seasons separately.

To test whether network structure contributes to species persistence, we also used a generalised linear mixed‐effects model with a beta‐error distribution. Here, we included fixed effects of restoration status, network structure (either nestedness or modularity), and their interaction, and included by‐site random intercepts. We conducted separate analyses for the month‐level and season‐level data. To account for the fact that species richness and number of interactions are correlated with nestedness and modularity, we also included a quadratic fixed effect for the total number of species and a linear effect of the total number of interactions.

Finally, to test the effect of centrality on species persistence, we fitted a linear mixed‐effects model with a binomial error distribution to the number of iterations (out of $10^{3}$) in which a species persisted. We included restoration status, species centrality, and their interaction as fixed effects, as well as by‐site, by‐species, and by‐network random intercepts. We ran separate models for weighted closeness and specialisation (d’), and separate analyses for the month‐level and season‐level data. Below we report both results separately.

For all the aforementioned analyses, we used the glmmTMB package (Brooks et al., 2017) in R (version 4.1; R Core Team, 2021). We fitted separate models for plants and pollinators. In the results below, we report Wald test statistics for the model parameters.

## RESULTS

**FIGURE 1:**
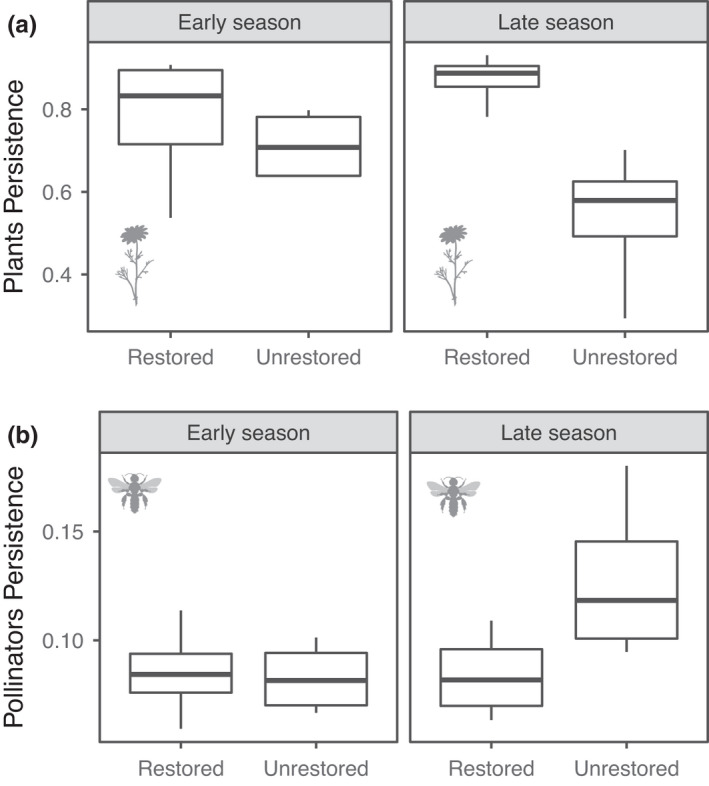
(a) Plant persistence was higher in restored sites, especially in late season whereas (b) pollinator persistence in the late season was higher in unrestored than in restored sites. Box plot represents median (mid line), interquartile range (box edges) and 1.5 × interquartile range (whiskers). Results shown for season‐level data

We expected that persistence of both plants and pollinators would be higher at restored sites, but we found contrasting support for this hypothesis. For the month‐level data, there was no clear effect of restoration on persistence of either plants or pollinators (Figure S1). However, for the season‐level data, we found that plant‐species persistence was higher in restored sites than in unrestored sites (restoration status: β=−1.02,p=0.002,df=11), and this effect was stronger in late season (restoration*season: β=−1.44,p<0.01,df=10), with plant persistence in restored sites being more than double that of unrestored sites (Figure 1a; Table S1). In contrast, for pollinators we found that persistence was marginally higher in the late season (β=0.24,p=0.07,df=11) and in unrestored sites (β=0.23,p=0.08,df=11), with a significant effect in late season (restoration*season: β=0.47,p=0.05,df=10; Figure 1b; Table S2). The interaction effect was confirmed when splitting the data by season, wherein treatment had no effect in pollinator persistence in the early season (β=−0.02,p=0.88,df=5; Table S2), but a clear effect in late season (β=0.44,p=0.02,df=5; Table S2).

**FIGURE 2:**
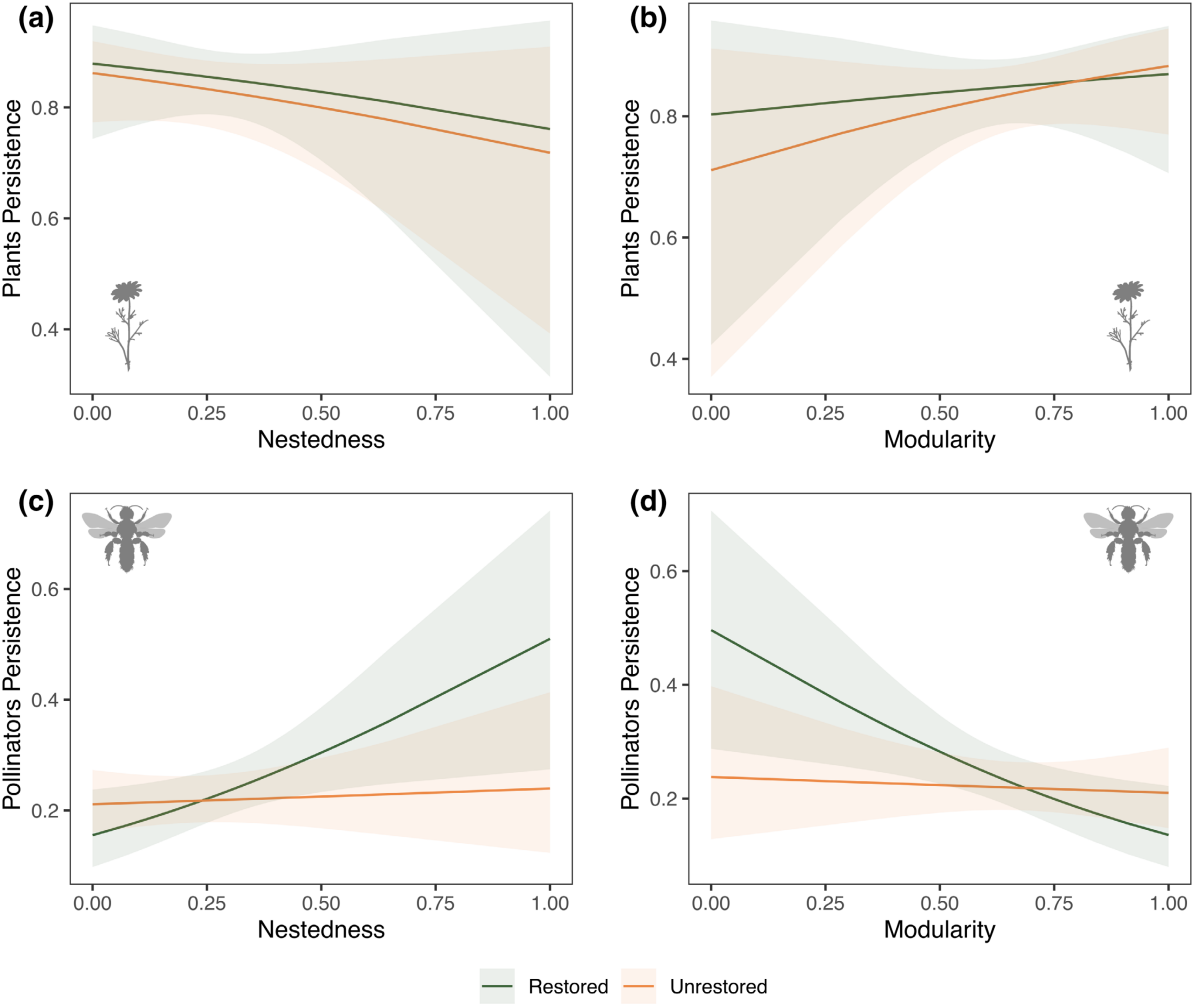
Neither nestedness (a) nor modularity (b) had an effect on plant persistence, whereas nestedness increased (c) and modularity decreased (d) pollinator persistence, and this effect was stronger in restored sites (green lines) than in unrestored sites (orange lines). Solid lines indicate the mean slope estimate from the GLMM model, and the shaded area represents the 95% confidence intervals around the estimate. Results shown for month‐level data

Given that previous work has shown that nestedness increases species persistence (Bastolla et al., 2009; Rohr et al., 2014; Thébault & Fontaine, 2010), we sought to explore whether restoration alters the association between network structure and persistence. For the month‐level data, we found that networks varied greatly in both their nestedness (range = 20.9−83.3; mean ± 1 SD; restored = 39.4±8.58; unrestored = 39.5±12.7) and modularity (range = 0.18−0.53; mean ± 1 SD; restored = 0.40±0.04; unrestored = 0.39±0.06). For plants, we found no effect of network structure on persistence (nestedness: β=−1.13,p=0.38,df=55; Figure 2a; Table S3; modularity: β=0.35,p=0.80,df=55; Figure 2b; Table S4), nor a support for a structure‐restoration interaction effect (nestedness: β=−0.34,p=0.83,df=55; modularity: β=0.79,p=0.59,df=55). In contrast, for pollinators, increases in nestedness resulted in greater pollinator persistence (β=1.73,p=0.02,df=55; Figure 2c; Table S5), whereas increases in modularity resulted in lower pollinator persistence (β=−1.84,p=0.01,df=55; Figure 2d; Table S6). We also found that restoration status modulated the effect of network structure on pollinator persistence. We found a clear interaction effect between restoration status and both nestedness (β=−1.57,p=0.050,df=55; Table S5) and modularity (β=1.67,p=0.03,df=55; Table S6), and this effect was stronger in unrestored sites (β=−1.15,p=0.02,df=55 Figure 2d). Conversely, at the season‐level data, we found no clear effect of network structure and persistence of either plants or pollinators.

**FIGURE 3:**
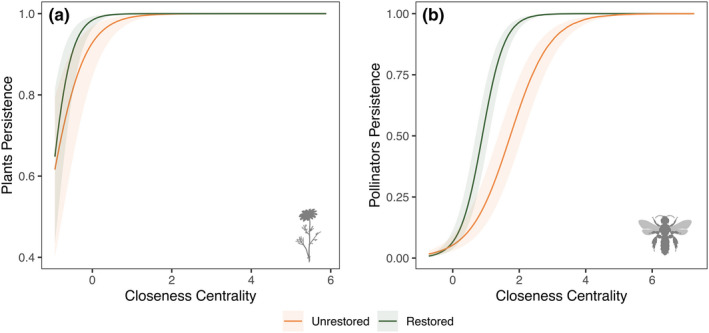
Species weighted closeness centrality had a clear, positive effect on mean persistence of both plants (a) and pollinators (b), and this effect was stronger in restored (green) than in unrestored (orange) sites. Weighted closeness centrality is scaled, solid line indicates the mean slope estimate from the GLMM model, and the shaded area represents the 95% confidence intervals around the estimate. Results shown for month‐level data

In addition to exploring persistence at the network‐level, we also investigated whether species that are more central in their interaction networks have greater persistence than more peripheral species, and whether there is a difference between restoration treatments. Here, we focus on the effect of weighted closeness, but we found similar results for specialisation (d’; Supporting Information). For the month‐level data, we found that centrality had a clear, positive effect on the persistence of both plants (β=3.73,p<0.001,df=428; Figure 3a; Table S7) and pollinators (β=2.98,p<0.001,df=818; Figure 3b; Table S8). We also found that the effect of centrality on persistence was weaker in unrestored sites, both for plants (restoration*centrality: β=−1.52,p<0.001,df=4298; Figure 3a; Table S7) and pollinators (restoration*centrality: β=−1.32,p<0.001,df=818; Figure 3b; Table S8). Likewise, we found the same pattern for the season‐level data, with centrality having a clear, positive effect on the persistence of both plants (early season: β=3.65,p<0.001,df=229; late season: β=8.14,p<0.001,df=226) and pollinators (early season: β=8.66,p<0.001,df=233; late season: β=4.60,p<0.001,df=202).

## DISCUSSION

Elucidating the interplay between ecological interactions and global change drivers such as the presence of invasive species is critical for anticipating how ecosystems respond to novel environmental scenarios (Blois et al., 2013; Traveset & Richardson, 2020). Our results highlight the importance of including species interactions and their community context when assessing the effects of restoration via invasive‐species removal on ecological communities (Cariveau et al., 2020; Memmott, 2009; Moreno‐Mateos et al., 2020; Traveset & Richardson, 2014). Specifically, we found that the removal of invasive plant species had a positive effect on the persistence of plant populations, especially in the late season, but the effect on pollinator populations was less clear. We found no effect of restoration on pollinator persistence in the early season, but pollinator persistence was highest in *unrestored* sites the late season. Moreover, pollinator persistence was more closely dependent on nestedness and modularity in restored sites than in unrestored sites, suggesting the existence of a link between the consequences of network structure and restoration status. Finally, at the species level, we found that more central species—both plants and pollinators—had a higher persistence on both restored and unrestored sites and that this effect was stronger in restored sites. We expand on each of these findings in the paragraphs below.

We expected that restoration via the removal of invasive plant species would benefit the native plants due to competition release. As expected, we found a clear positive effect of restoration status in plant persistence, especially in the late season, with plants in restored sites having greater persistence. Originally, Kaiser‐Bunbury et al. (2017) found that plant species in restored sites had higher visitation frequency, resulting in higher fruit sets. It is reasonable to assume that this leads to greater plant persistence in restored sites, as suggested by our results. In contrast, for animals, we found that the effect of restoration varied between seasons. While pollinator persistence was similar across treatments in the early season, in the late season the effect of restoration varied, with pollinators having greater persistence in unrestored sites. We propose two complementary hypotheses for this counter‐intuitive result. First, given that the removed invasive plant species were dominant and widespread (Kaiser‐Bunbury et al., 2017), the fact that exotic plant species are generally abundant in invaded systems (Albrecht et al., 2014; Traveset & Richardson, 2014, 2020), and that alien plants may increase primary production in invaded ecosystems (Vilà et al., 2011), the exotic plants removed may represent important resources for pollinators (Bartomeus et al., 2016). For example, one of the exotic species removed, *Cinnamomum verum*, is known to have long flowering seasons (from November until March; Azad et al., 2018) and flowers two to three times a year (Yulistyarini, 2020). Thus, even though introduced species may compromise the pollination of native plants (Russell & Kaiser‐Bunbury, 2019; Traveset & Richardson, 2014), they may represent important resources to pollinators (Arroyo‐Correa et al., 2020), especially in ecosystems with marked seasonality, which could explain the increased persistence of pollinators in the unrestored sites in the late season.

Another potential explanation for greater pollinator persistence in unrestored sites may lie in the long‐term response of native species in the restored areas. In a similar restoration experiment, Heleno et al. (2010) found that the number of seeds produced by the focal native species was much higher in the second year after restoration. Given that the data we used were collected in the first flowering season following restoration (Kaiser‐Bunbury et al., 2017), we can hypothesise the existence of a delayed restoration benefit for pollinators, mediated by a response of native plants after being disturbed by the removal of invasive and exotic species during the restoration process. Our results indicate that an unexpected result of invasive plant removal could be a short‐term loss of late‐season resources for pollinators, at least in the first flowering season following restoration. Given the importance of plant phenology for wild pollinators (Timberlake et al., 2019), managers should thus be aware of potential hidden effects on non‐target species in invaded communities following restoration interventions such as invasive‐species removal (Traveset & Richardson, 2020). Taken together, our findings suggest the importance of considering different restoration strategies when attempting to restore interactions, rather than species per se (Devoto et al., 2012; Gawecka & Bascompte, 2021). For example, after community restoration via invasive species removal, it may be necessary to also include extra resources for pollinators. By focusing mainly on the response of native plants to restoration, we may be overlooking pollinator survival in the short term.

We also explored whether there was an effect of community structure on the persistence of plants and pollinators. For the season‐level data, we found no clear effect of network structure and persistence of either plants or pollinators. However, this is unsurprising given that analysing networks at finer temporal scales better identify nuances in network properties (CaraDonna & Waser, 2020). In contrast, as expected from other studies (Bastolla et al., 2009; Lever et al., 2020; Rohr et al., 2014; Thébault & Fontaine, 2010), we found that higher nestedness was related to greater pollinator persistence, whereas modularity decreased pollinator persistence. Interestingly, the effect sizes of both nestedness and modularity varied between restoration treatments, with only restored sites having significant effects. Therefore, although many studies have focused on the effect of network structure on species persistence, we found this association to be much stronger in restored ecosystems. The fact that pollinators' persistence was higher at unrestored sites suggests that restoration may enforce the structure‐persistence relationship on pollinators whereas the resources provided by invasive plants may have uncoupled the association, allowing for high pollinator persistence even in less nested networks.

In addition to looking at species persistence from the perspective of the whole community structure, we also explored whether the position of specific species in their networks could be related to their persistence. In an experimental approach using centrality in restoration practices, Maia et al. (2019) found that planting plants with high network centrality in restoration plots attracted a higher abundance and richness of pollinators than planting peripheral plant species. Combined with our findings that centrality has a clear positive effect on species persistence, these results suggest that incorporating species‐level network metrics in restoration practices may increase long‐term restoration effectiveness while also accelerating restoration at the landscape level (Moreno‐Mateos et al., 2020). Given that interactions disappear faster than species themselves (Janzen, 1971), if the goal of restoration is to conserve species and ecosystem functions in the long term (Brudvig, 2011; Clewell & Aronson, 2012; Traveset & Richardson, 2020), it is necessary to ensure the maintenance of the structure of ecological networks to guarantee the persistence of entire communities. Our results add to previous studies highlighting that network structure can be used as a proxy not only for the provisioning of ecosystem services (Kaiser‐Bunbury et al., 2017; Traveset & Richardson, 2020), but also in restoration studies to assess the impact of restoration practices on population dynamics (Gaiarsa et al., 2021; Tylianakis et al., 2010).

In this work, we explored the effects of community structure on species persistence after restoration. Although we acknowledge that neutral‐processes play a role in the observed changes in species persistence, recent empirical work in other systems has demonstrated the importance of niche‐based processes in determining biotic interactions. In a seed‐dispersal network study in the highly invaded Oahu Island in Hawaii, Vizentin‐Bugoni et al. (2021) showed that invasive‐plant species interactions were driven primarily through plants' seed size than through plants abundance, while in an agricultural landscape, Kremen et al. (2018) found that early in the restoration process floral diversity was more important to native pollinators than floral density. Likewise, in another study, both richness and abundance of native pollinators were higher in restored than in unrestored grassland patches, despite plant abundance being similar across treatments (Noreika et al., 2019). Thus, although patterns in community structure may be influenced by neutral processes (Fründ et al., 2016), given that ecological interactions and the resulting network patterns also influence species' demography (Xi et al., 2020), the effect of abundance on the observed network structure is not unidirectional (Guimarães Jr, 2020). These and other studies indicate the importance of ecological traits and niche‐based processes as mediators of species persistence and the effectiveness of restoration.

The ultimate success of restoration strategies is the persistence of wild populations (Miller et al., 2017), but there are only a handful of studies connecting populations to restoration practices (e.g., Gaiarsa et al., 2021; Rodewald et al., 2015). For restoration to be successful, it is necessary to assess not only how ecosystem functions, such as pollination, are impacted by restoration practices (Cariveau et al., 2020; Kaiser‐Bunbury et al., 2017; Miller et al., 2017), but also how the remaining species are affected in the time following restoration. Our results emphasise hidden, contrasting effects of species invasions—while invasive species can prune ecological networks through competition with native species, invasive plant species may also have a positive effect on the animals in the community. We suggest that to plan successful restoration practices, it may be important to also account for hidden effects resulting from the removal of exotic species (Emer & Timóteo, 2020) in order to decrease the potential for indirect detrimental effects on the remaining species, which can also include population assessments before and after restoration interventions to increase restoration effectiveness in the long‐term (Moreno‐Mateos et al., 2020; Suding et al., 2015). We show that exotic species may not only affect species abundance and diversity while present, but their effects may be pervasive even after their removal, potentially hampering the ability of ecological communities to recover from additional perturbations. We hope that our approach incorporating interaction dynamics in restoration practices may help predict future trajectories of ecological communities and help ensure the long‐term functioning of the web of life.

## AUTHORSHIP

MPG and JB designed the study. MPG collected data, performed analyses, and prepared the figures, with input from JB. MPG wrote the manuscript and JB contributed to revisions.

## Supporting information


Supporting information S1

## Data Availability

Data is available from the original paper and analyses code is deposited in Zenodo (https://doi.org/10.5281/zenodo.6832392).
