# Supplementary material for: Hidden effects of habitat restoration on the persistence of pollination networks
Source: Ecol Lett. 2022 Aug 25;25(10):2132–41. doi: 10.1111/ele.14081 (PMC9804604; doi:10.1111/ele.14081)
Supplement: Supplementary file 1 — Supporting information S1 [file ELE-25-2132-s001.pdf]

Online Supplement

Hidden effects of habitat restoration on the persistence of pollination networks

Marilia P. Gaiarsa & Jordi Bascompte

Ecology Letters

Supplementary Figures and Tables

1) Monthly-level analysis

We expected that persistence of both plants and pollinators would be higher at restored sites. However, for the month-level data, there was no clear effect of restoration on persistence of neither plants ($\beta = 0.28, p = 0.54, df = 58$; Fig. S1a), nor pollinators ($\beta = 0.09, p = 0.22, df = 58$; Fig. S1b).

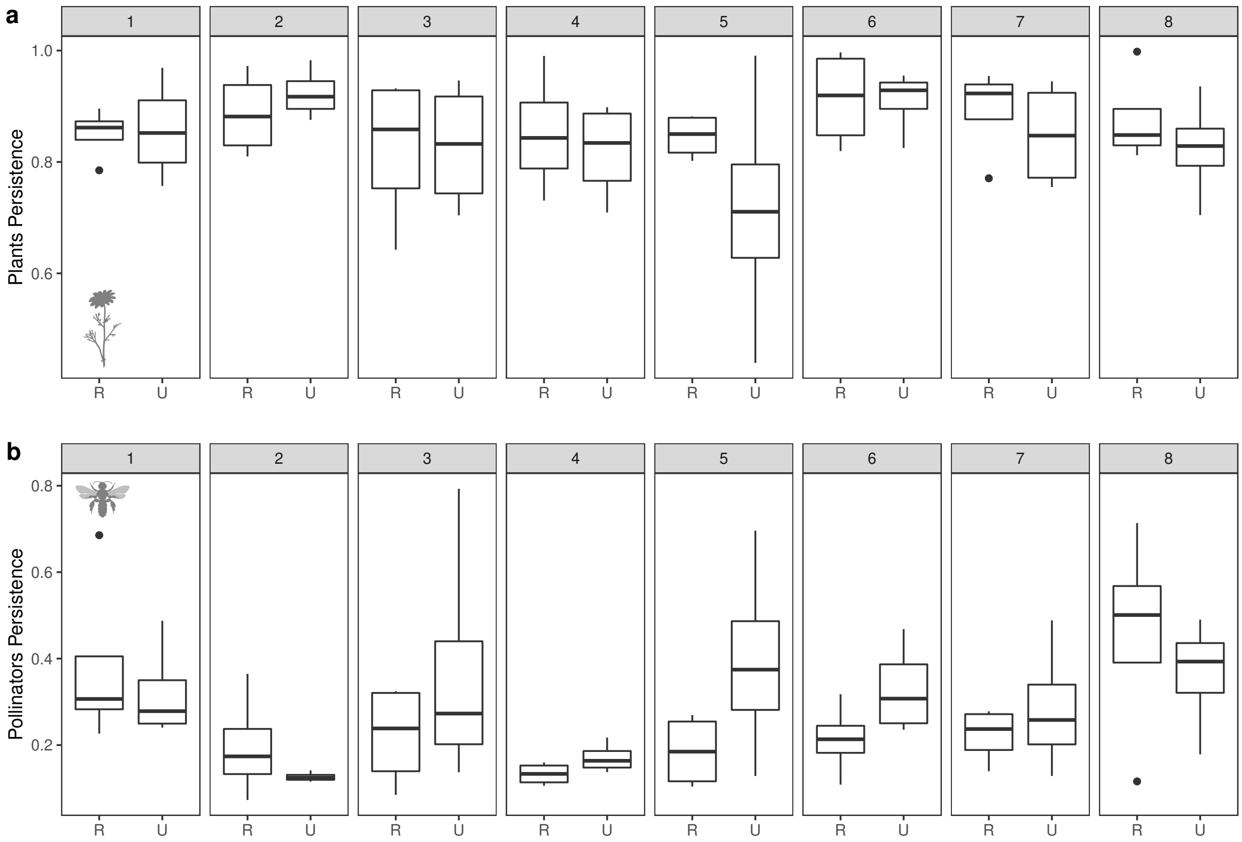

Figure S1: Invasive plant species removal had no effect on population persistence between restored (R) and unrestored (U) sites, neither for plants (a), nor pollinators (b), at each different sampling month (numbers on the top). Box plot represent median (mid line), interquartile range (box edges), and 1.5 x interquartile range (whiskers). Equivalent to Fig. 1 in main text but with the monthly-level data (as opposed to early and late season). Bee image by Melissa Broussard, CC BY 3.0, courtesy of PhyloPic.org.

2) Effect of specialization (d′) on species persistence

For the month-level data we found that specialization had a clear, positive effect on the persistence of both plants ($\beta = 0.23, p < 0.001, df = 426$; Fig. S2a) and pollinators ($\beta = 0.23, p < 0.001, df = 816$; Fig. S2b). We also found that the effect of specialization on persistence was weaker in unrestored sites, both for plants (restoration*specialization: $\beta = -0.13, p < 0.001, df = 426$; Fig. S2a) and pollinators (restoration*specialization: $\beta = -0.02, p = 0.003, df = 816$; Fig. S2b).

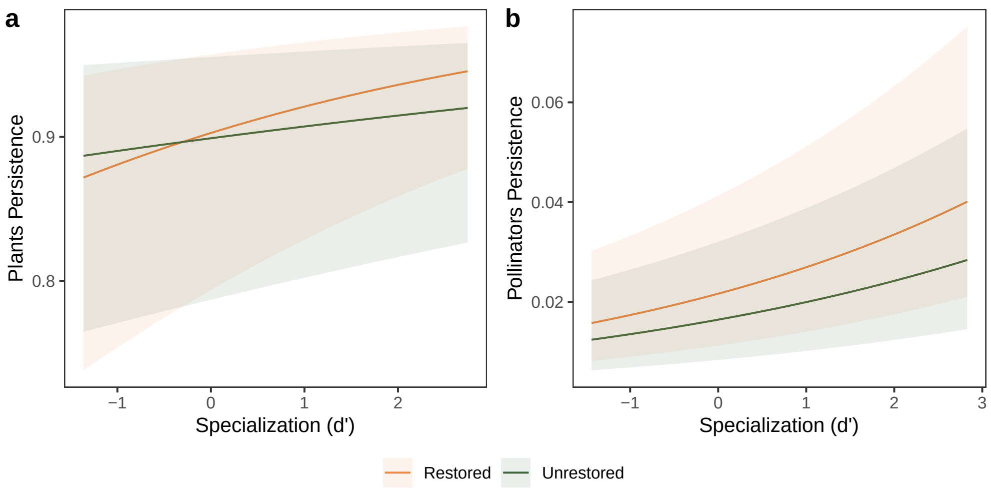

Figure S2: Species specialization (d′) had a clear, positive effect on mean persistence of both plants (a) and pollinators (b), and this effect was stronger in restored (green) than in unrestored (orange) sites. Specialization is scaled, solid line indicates the mean slope estimate from the GLMM model, and the shaded area represents the 95% confidence intervals around the estimate. Results shown for month-level data.

We found similar patterns for the season-level data, with specialization having a positive effect on the persistence of both plants ($\beta = 1.47, p < 0.001, df = 457$) and pollinators (early season: $\beta = 0.43, p < 0.001, df = 437$), and that the effect of specialization varied across treatments (plants restoration*specialization: $\beta = -1.03, p < 0.001, df = 457$; pollinators restoration*specialization: $\beta = 0.20, p = 0.003, df = 437$).

3) Analysis including vertebrates

We omitted vertebrate species from the analysis in the main text because of the order-of-magnitude differences in invertebrate and vertebrate longevity, and the model assumption that animals (pollinators) are completely dependent on plants for their survival, which may not be true for the five vertebrate species present in the original dataset (Kaiser-Bunbury *et al.*, 2017). Vertebrate species include two species of birds, *Cinnyris dussumieri* (a nectarivore and insectivorous sunbird) and *Hypsipetes crassirostris* (a bulbul species that feeds on fruits and flowers as well as arthropods and bird eggs), and also three species of lizards, *Hemidactylus frenatus*, *Mabuya seychellensis*, and *Phelsuma* sp., all of which are primarily insectivorous. In the figures below we show that results were qualitatively the same to the ones presented in the main text (Figs. S3, S4, S5, S6), regardless of the inclusion of vertebrates.

3.1) Effect of month and season on species persistence

As in the analyses in the main text, we used generalized linear mixed-effects models to test the effect of restoration status on species persistence. We included month, restoration status, and their interaction as fixed effects and included by-site random intercepts. We assumed a beta-error distribution. We used a similar model to test for effects of restoration status and seasonality on species persistence, swapping the fixed effects of month and month-restoration interaction for fixed effects of season and a season-restoration interaction.

We expected that persistence of both plants and pollinators would be higher at restored sites, but we found contrasting support for this hypothesis. For the month-level data, there was no clear effect of restoration on persistence of neither plants ($\beta = 0.31, p = 0.48, df = 58$; Fig. S3a) nor pollinators ($\beta = 0.04, p = 0.89, df = 58$; Fig. S3b). However, for the season-level data, we found that plant persistence was lower in unrestored sites ($\beta = -1.02, p = 0.005, df = 10$), especially in the late season (restoration*season: $\beta = -1.19, p = 0.009, df = 10$; Fig. S4a) whereas pollinator persistence in the late season was higher ($\beta = 0.30, p = 0.09, df = 10$; Fig. S4b), but there was no interaction effect between restoration and season.

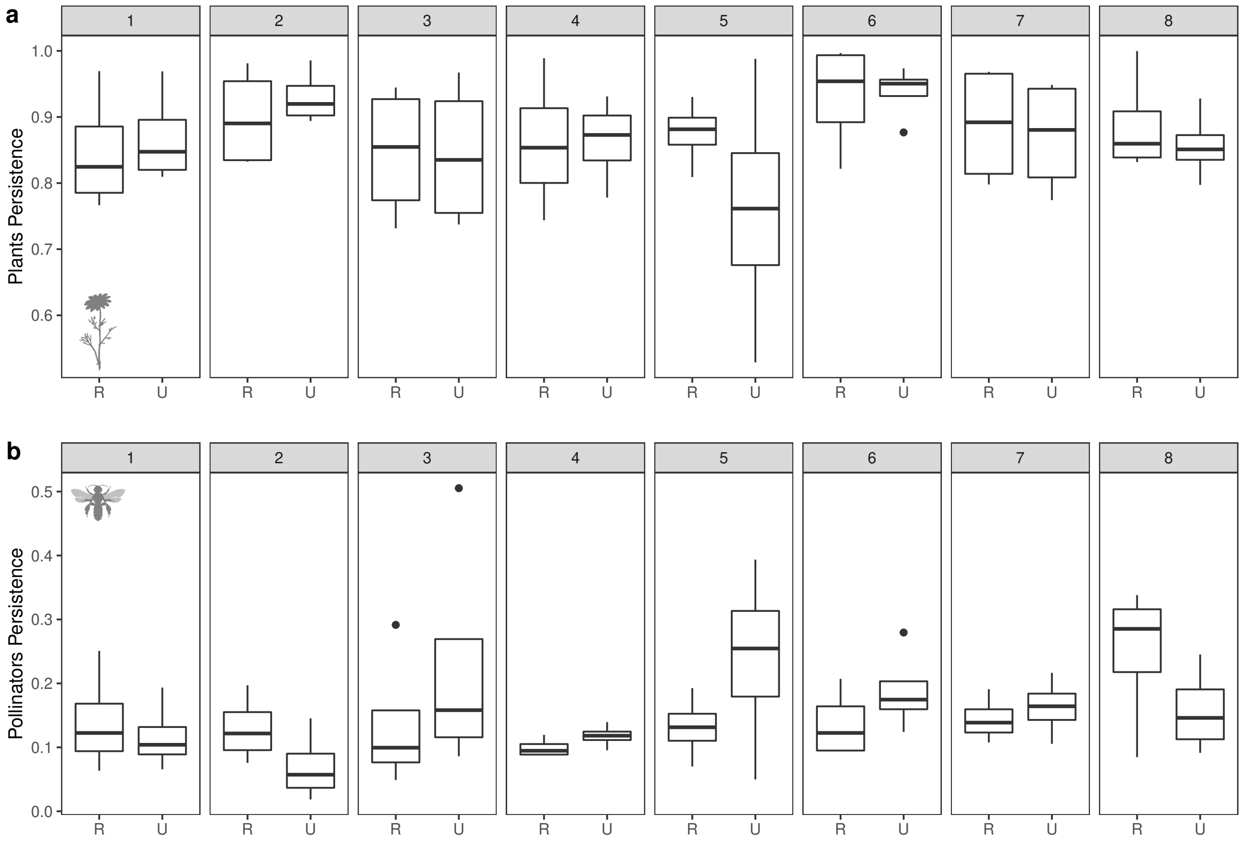

Figure S3: Invasive plant species removal had no effect on (a) plants, nor (b) pollinators persistence. Box plot represent median (mid line), interquartile range (box edges), and 1.5 x interquartile range (whiskers). Equivalent to Fig. S1 but including vertebrates. Bee image by Melissa Broussard, CC BY 3.0, courtesy of PhyloPic.org.

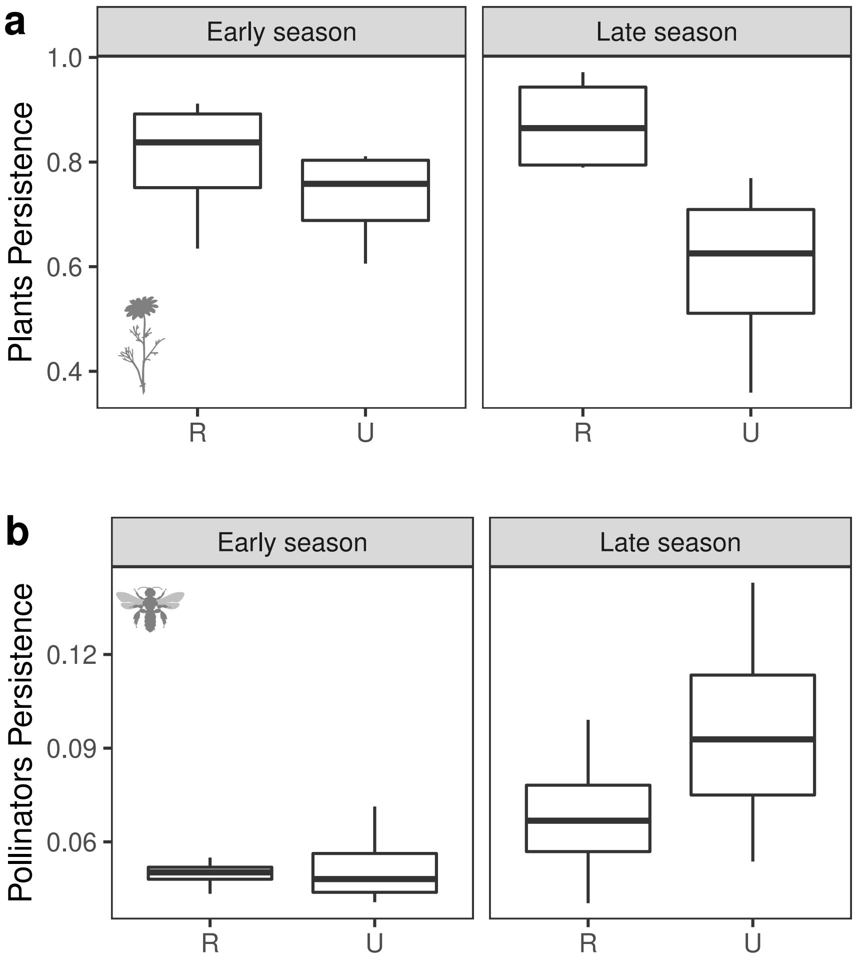

Figure S4: (a) Plant persistence was higher in restored sites, especially in late season whereas (b) pollinator persistence was higher in the late season, especially in unrestored sites. Box plot represent median (mid line), interquartile range (box edges), and 1.5 x interquartile range (whiskers). Equivalent to Fig. 1 in main text but including vertebrates. Bee image by Melissa Broussard, CC BY 3.0, courtesy of PhyloPic.org.

3.2) Effect of network structure on species persistence

To test whether nestedness and modularity contribute to species persistence, we used a generalized linear mixed-effects model with a beta-error distribution, including fixed effects of restoration status, network structure (either nestedness or modularity), and their interaction. We fit by-site random intercepts. To account for the fact that species richness is correlated with nestedness and modularity, we also included a quadratic fixed effect for the total number of species.

For the month-level data, we found no effect of network structure on plant persistence for neither nestedness ($\beta = -0.02, p = 0.20, df = 55$; Fig. S5a), nor modularity ($\beta = 3.98, p = 0.46, df = 55$; Fig. S5b), nor a support for a structure-restoration interaction for nestedness ($\beta = 0.03, p = 0.18, df = 55$) nor modularity ($\beta = -4.71, p = 0.38, df = 55$). In contrast, for pollinators, increases in nestedness resulted in a strong, positive effect on pollinator persistence ($\beta = 0.02, p = 0.009, df = 55$; Fig. S5c), whereas increases in modularity were not significant ($\beta = -2.95, p = 0.12, df = 55$; Fig. S5d). Similarly to results presented in the main text, for the season-level data, there was no clear effect of network structure on the persistence of plants, whereas nestedness had a weak effect on pollinator persistence ($\beta = -0.07, p = 0.01$), but modularity had no effect.

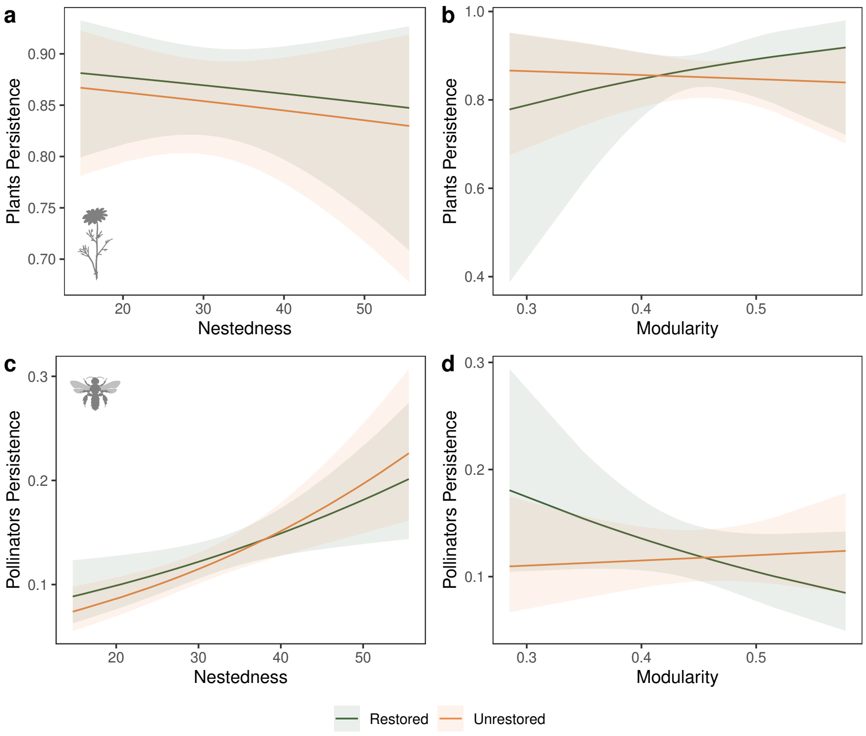

Figure S5: Nestedness (a) and modularity (b) and no effect on plant persistence. In contrast, nestedness had a strong positive effect on pollinator persistence (c) whereas modularity had no effect on pollinator persistence (d). Solid lines indicate the mean slope estimate from the GLMM model, and the shaded area represents the 95% confidence intervals around the estimate. Equivalent to Fig. 2 in main text but including vertebrates. Results shown for month-level data. Bee image by Melissa Broussard, CC BY 3.0, courtesy of PhyloPic.org.

3.3) Effect of centrality on species persistence

To test whether species that are more central in their interaction networks have greater persistence than more peripheral species, we fitted a binomial linear mixed-effects model to the number of iterations (out of 10^3) in which a species persisted. We included restoration status, species centrality, and their interaction as fixed effects, as well as by-site and by-species random intercepts. We found that network centrality had a positive effect on the persistence of both plants ($\beta = 3.82, p < 0.001, df = 434$; Fig. S6a) and pollinators ($\beta = 2.28, p < 0.001, df = 1184$; Fig. S6b). Moreover, the effect of centrality on persistence was weaker in unrestored sites, both for plants ($\beta = -1.52, p < 0.001$) and pollinators ($\beta = -0.54, p < 0.001$). We found similar results for the season-level data, with centrality having a clear, positive effect on the persistence of both plants (early season: $\beta = 4.75, p < 0.001, df = 231$; late season: $\beta = 9.85, p < 0.001, df = 227$) and pollinators (early season: $\beta = 8.57, p < 0.001, df = 330$; late season: $\beta = 2.35, p < 0.001, df = 290$).

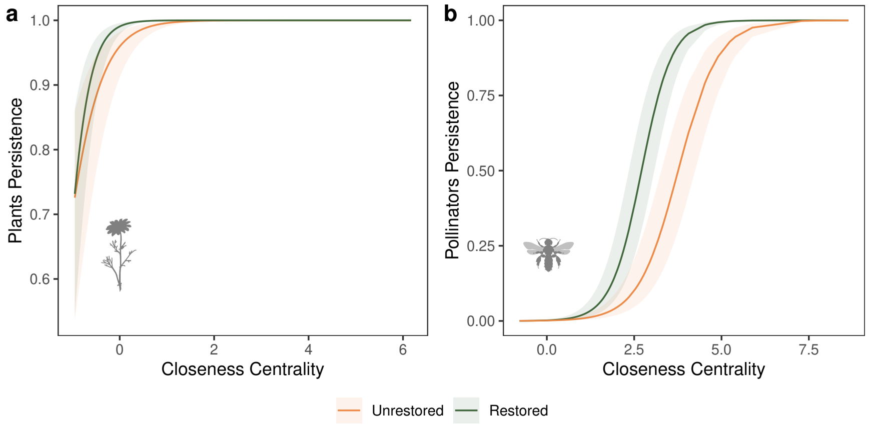

Figure S6: Species network centrality had a positive effect on the persistence of both plants (a) and pollinators (b). Centrality is scaled, solid line indicates the mean slope estimate from the GLMM model, and the shaded area represents the 95% confidence intervals around the estimate. Equivalent to Fig. 3 in main text but including vertebrates. Bee image by Melissa Broussard, CC BY 3.0, courtesy of PhyloPic.org.

4) Exploring different parameter values for the dynamic model

In this section we show that our results remain the same after exploring different, broader, parameter values from the main text (based on previous studies; Bastolla *et al.* 2009; Lever *et al.* 2020; Saavedra *et al.* 2011). In the figures below (Figs. S7, S8, S9) we used the following parameter values: intrinsic growth rates α_i were drawn uniformly from the interval $[0.75, 1.2]$; values for intraspecific competition β_{ii} were drawn uniformly from the intervals $[0.97, 1.03]$ and those for interspecific competition β_{ij} from $[0.20, 0.26]$; the per capita effects of mutualistic interaction, γ were drawn uniformly from the interval $[0.16, 0.24]$; handling time h was fixed and set to 0.1; initial abundance densities S were drawn uniformly from the interval $(0, 1]$.

4.1) Effect of season on persistence

We found that plant persistence was higher in late season ($\beta = 0.62, p = 0.02, df = 10$), and that this effect was stronger in restored sites than in unrestored sites ($\beta = -1.49, p < 0.001, df = 10$; Fig. S7a). In contrast, for pollinators we found that persistence was higher in the late season in unrestored sites (restoration*season: $\beta = 0.49, p = 0.05, df = 10$; Fig. S7b).

4.2) Effect of network structure on persistence

For plants, we found no effect of network structure on persistence (nestedness: $\beta = -0.02, p = 0.33, df = 55$; modularity: $\beta = 0.67, p = 0.86, df = 55$; Fig. S8a & b) nor a support for a structure-restoration interaction ($\beta = -0.001, p = 0.96, df = 55$; $\beta = 4.32, p = 0.31, df = 55$). In contrast, for pollinators, increases in nestedness resulted in greater pollinator persistence ($\beta = 0.02, p = 0.04, df = 55$; Fig. S8c), whereas increases in modularity resulted in lower pollinator persistence ($\beta = -4.96, p = 0.01, df = 55$; Fig. S8d). We found a clear interaction effect between restoration status and both nestedness ($\beta = -0.023, p = 0.07, df = 55$) and modularity ($\beta = 5.23, p = 0.01, df = 55$), with pollinators in unrestored sites being less persistent than expected from their structure compared to restored sites (Fig. S8c & d).

4.2) Effect of centrality on species persistence

We found that weighted closeness centrality had a clear, positive effect on the persistence of both plants ($\beta = 0.06, p < 0.001, df = 428$; Fig. S9a) and pollinators ($\beta = 0.34, p < 0.001, df = 575$; Fig. S9b). We also found that the effect of centrality on persistence was weaker in unrestored sites for pollinators (restoration*centrality: $\beta = -0.18, p < 0.001, df = 575$; Fig. S9b).

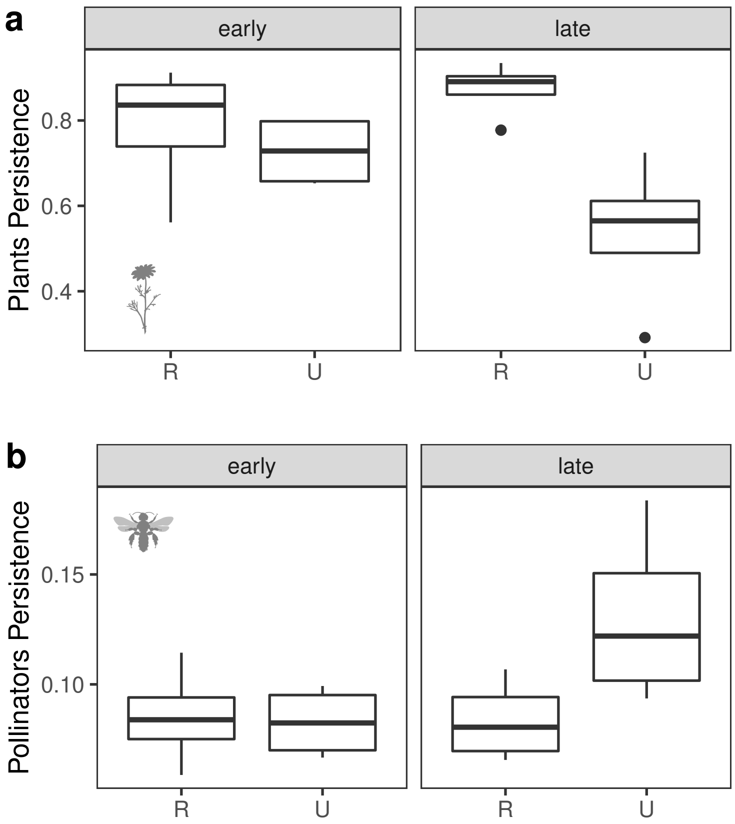

Figure S7: (a) Plant persistence was higher in restored sites in late season whereas (b) pollinator persistence in the late season was higher in unrestored than in restored sites. Box plot represent median (mid line), interquartile range (box edges), and 1.5 x interquartile range (whiskers). Equivalent to Fig. 1 in main text but with a broader range of parameter values. Bee image by Melissa Broussard, CC BY 3.0, courtesy of PhyloPic.org.

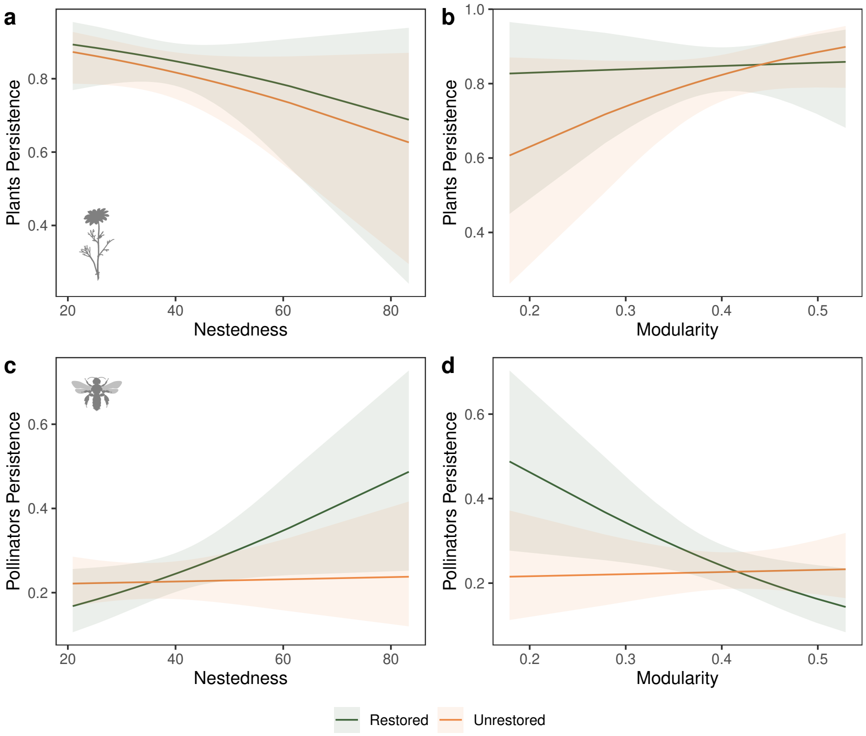

Figure S8: (a) Network nestedness and (b) modularity had no effect on plant persistence. In contrast, for pollinators (c) nestedness increased and (d) modularity decreased pollinator persistence. Solid lines indicate the mean slope estimate from the GLMM model, and the shaded area represents the 95% confidence intervals around the estimate. Equivalent to Fig. 2 in main text but with a broader range of parameter values. Bee image by Melissa Broussard, CC BY 3.0, courtesy of PhyloPic.org.

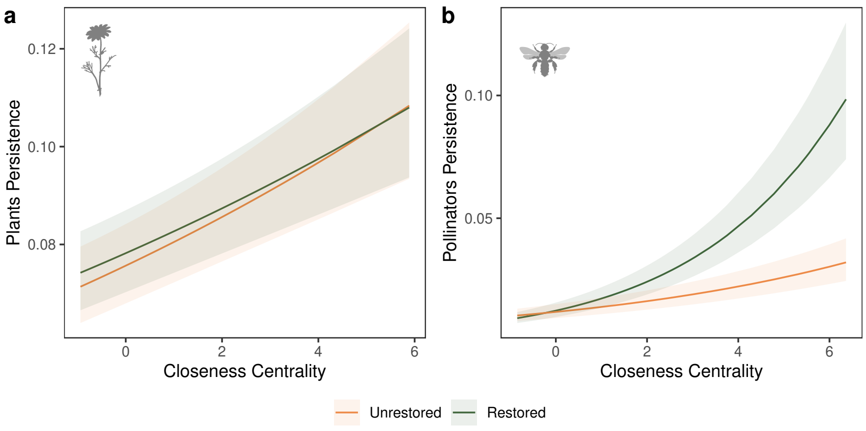

Figure S9: Species weighted closeness centrality had a positive effect on the persistence of both (a) plants and (b) pollinators. Centrality is scaled, solid line indicates the mean slope estimate from the GLMM model, and the shaded area represents the 95% confidence intervals around the estimate. Equivalent to Fig. 3 in main text but with a broader range of parameter values. Bee image by Melissa Broussard, CC BY 3.0, courtesy of PhyloPic.org.

5) Tables of statistical results presented in the main text

5.1) Effect of seasonality on species persistence

Table S1: Effect of seasonality on plant persistence. Model includes a random effect of site; $df = 10$.

	Estimate	Std. Error	z value	Pr(>|z|)
(Intercept)	1.349	0.309	4.361	0.000
late season	0.656	0.270	2.431	0.015
TreatmentUnrestored	-0.405	0.433	-0.935	0.350
late season:TreatmentU	-1.446	0.344	-4.202	0.000

Table S2: Effect of seasonality on pollinator persistence. GLLM includes a random effect of site; $df_{GLLM} = 10$, $df_{LM} = 5$.

	Estimate	Std. Error	z value	Pr(>|z|)
**GLLM (season*treatment)**				
(Intercept)	-2.371	0.130	-18.222	0.000
late season	-0.015	0.178	-0.082	0.935
TreatmentU	-0.022	0.182	-0.119	0.905
late season:TreatmentU	0.474	0.245	1.938	0.053
**Linear model (early season)**				
(Intercept)	-2.377	0.109	-21.704	0.000
TreatmentU	-0.023	0.155	-0.147	0.883
**Linear model (late season)**				
(Intercept)	-2.375	0.146	-16.271	0.000
TreatmentU	0.446	0.190	2.353	0.019

5.2) Effect of network structure on species persistence

Table S3: Effect of Nestedness on Plants (monthly data). Model includes a random effect of site; $df = 55$.

	Estimate	Std. Error	z value	Pr(>|z|)
(Intercept)	-0.118	1.457	-0.081	0.936
nodf	-1.134	1.439	-0.788	0.430
TreatmentUnrestored	-0.216	0.546	-0.395	0.693
poly(log(TotalSpp), 2)1	-2.621	1.604	-1.633	0.102
poly(log(TotalSpp), 2)2	1.000	0.891	1.123	0.262
log(Nints)	0.479	0.315	1.521	0.128
nodf:TreatmentUnrestored	0.340	1.585	0.214	0.830

Table S4: Effect of Modularity on Plants (monthly data). Model includes a random effect of site; $df = 55$.

	Estimate	Std. Error	z value	Pr(>|z|)
(Intercept)	-0.365	2.126	-0.172	0.864
mod	0.346	1.363	0.254	0.799
TreatmentUnrestored	-0.593	0.986	-0.601	0.548
poly(log(TotalSpp), 2)1	-2.303	1.646	-1.399	0.162
poly(log(TotalSpp), 2)2	1.309	0.895	1.461	0.144
log(Nints)	0.416	0.315	1.322	0.186
mod:TreatmentUnrestored	0.792	1.496	0.529	0.597

Table S5: Effect of Nestedness on Pollinators (monthly data). Model includes a random effect of site; $df = 55$.

	Estimate	Std. Error	z value	Pr(>|z|)
(Intercept)	-1.763	0.290	-6.076	0.000
nodf	1.732	0.736	2.353	0.019
TreatmentUnrestored	0.375	0.312	1.204	0.229
poly(log(TotalSpp), 2)1	-5.515	0.804	-6.860	0.000
poly(log(TotalSpp), 2)2	0.375	0.538	0.697	0.486
Nints	0.002	0.001	1.483	0.138
nodf:TreatmentUnrestored	-1.569	0.798	-1.965	0.049

Table S6: Effect of Modularity on Pollinators (monthly data). Model includes a random effect of site; $df = 55$.

	Estimate	Std. Error	z value	Pr(>|z|)
(Intercept)	-0.070	0.515	-0.136	0.892
mod	-1.836	0.713	-2.574	0.010
TreatmentUnrestored	-1.150	0.480	-2.396	0.017
poly(log(TotalSpp), 2)1	-5.415	0.831	-6.516	0.000
poly(log(TotalSpp), 2)2	0.323	0.521	0.621	0.535
Nints	0.001	0.001	1.321	0.186
mod:TreatmentUnrestored	1.676	0.747	2.244	0.025

5.3) Effect of centrality on species persistence

Table S7: Effect of Centrality on Plants (monthly data). Weighted closeness centrality is scaled and model includes random effects of site, species, and network; $df = 428$.

	Estimate	Std. Error	z value	Pr(>|z|)
(Intercept)	4.119	0.446	9.237	0.000
TreatmentUnrestored	-1.563	0.553	-2.828	0.005
close.scale	3.729	0.037	100.218	0.000
TreatmentUnrestored:close.scale	-1.515	0.045	-33.945	0.000

Table S8: Effect of Centrality on Pollinators (monthly data). Weighted closeness centrality is scaled and model includes random effects of site, species, and network; $df = 818$.

	Estimate	Std. Error	z value	Pr(>|z|)
(Intercept)	-2.645	0.305	-8.672	0.000
TreatmentUnrestored	-0.230	0.378	-0.609	0.543
close.scale	2.978	0.017	175.372	0.000
TreatmentUnrestored:close.scale	-1.317	0.020	-65.098	0.000

References

Bastolla, U., Fortuna, M.A., Pascual-García, A., Ferrera, A., Luque, B. & Bascompte, J. (2009). The architecture of mutualistic networks minimizes competition and increases biodiversity. *Nature*, 458, 1018–1020.

Kaiser-Bunbury, C.N., Mougal, J., Whittington, A.E., Valentin, T., Gabriel, R., Olesen, J.M. & Blüthgen, N. (2017). Ecosystem restoration strengthens pollination network resilience and function. *Nature*, 542, 223–227.

Lever, J.J., van de Leemput, I.A., Weinans, E., Quax, R., Dakos, V., van Nes, E.H., Bascompte, J. & Scheffer, M. (2020). Foreseeing the future of mutualistic communities beyond collapse. *Ecology Letters*, 23, 2–15.

Saavedra, S., Stouffer, D.B., Uzzi, B. & Bascompte, J. (2011). Strong contributors to network persistence are the most vulnerable to extinction. *Nature*, 478, 233–235.
